# Obstetric fistulas in Uganda: scoping review using a determinant of health approach to provide a framework for health policy improvement

**DOI:** 10.1186/s12884-020-02951-7

**Published:** 2020-04-29

**Authors:** Geerte C. den Hollander, Erica W. M. Janszen

**Affiliations:** 1 Maternity and Surgical Departments, Saint Francis Hospital, Mutolere, Kisoro Municipality Council, Kisoro, Uganda; 2 Gynaecology and Obstetrics Department, Kampala Hospital, 6C Makindu Close, Kololo, Kampala, Uganda; 3 Gynaecology and Obstetrics Department, OLVG Hospital, location Oost, Oosterpark 9, Amsterdam, the Netherlands

**Keywords:** Obstetric fistula, Maternal health, Determinants of health, Health policy, Inequity, Poverty, Uganda

## Abstract

**Background:**

The uneven global and national distribution of obstetric fistulas suggests a complex network of determinants contributing to fistula development. This study aims to create an understanding of the determinants of obstetric fistula in Uganda and to give a framework for health policy improvement.

**Methods:**

A scoping review of existing literature was performed, searching the PubMed/MEDLINE database, Ugandan Demographic and Health Surveys, and official sources of Ugandan statistics. Data was analysed using the model for the determinants of health by Dahlgren and Whitehead.

**Results:**

Obstetric fistulas are associated with different personal lifestyle factors, certain social and community networks, as well as poor working and living conditions. Malnutrition, early childbearing, limited female empowerment, lack of awareness of childbearing risks, low socioeconomic status, and long distances to emergency obstetric care play a part. Certain regions of Uganda are in particular associated with obstetric fistula, where an accumulation of determinants is notable.

**Conclusion:**

Analysis using the model of Dahlgren and Whitehead shows that obstetric fistulas are associated with determinants at different levels of society. Poverty and low education link these in a web that is disproportionately hard to escape from for the poorest women. This inequity asks for co-operation between ministries to dismantle the environment for obstetric fistula.

## Synopsis

Obstetric fistulas are associated with severe inequity on multiple levels. Analysis using the determinant of health approach provides targets for intervention on those levels.

## Background

It has been called a “global social injustice” [1], the “result of the egregious failure of health systems” [2] and even a “human rights tragedy” [3]. While describing the burden of obstetric fistulas (OF) the use of superlatives is not feared. As a complication of obstructed labour, an OF is an abnormal connection between the vagina and the bladder or the rectum. This leaves the woman incontinent for urine, faeces, or even both. Besides the physical consequences of the constant leakage of bodily fluids, the associated foul smell and psychological stigmatization have enormous social and economic consequences, as well as devastating emotional and psychological consequences, that include shame, loss of self-esteem, depression, rejection, and loss of marital and sexual rights [4–6].

The World Health Organization (WHO) estimates that worldwide between 50.000–100.000 women develop OF each year and 2 million women live with untreated fistula in Asia and sub-Sahara Africa (SSA), but the exact number remains difficult to measure [7, 8]. Within SSA, Uganda has the highest known prevalence of OF with an estimated lifetime prevalence of 19,2 per 1000 women of reproductive age [9]. The data of the last two Demographic and Health Surveys (DHS) show that 1 to 2% of Ugandan women have symptoms of OF, of whom only 62% have sought treatment [10, 11]. Those not seeking treatment give as reason being too embarrassed to seek treatment, while others did not know where to find treatment [10]. Furthermore, it is believed that even if all affected women would seek treatment, at the current rate that fistulas are surgically being managed, it will take at least 55 years to treat all existing patients, let alone to treat the new cases that develop every year [2].

According to WHO most cases of fistula can be avoided, firstly by delaying the age of first pregnancy and secondly by access to obstetric care [7]. Fistulas have been practically eliminated in many countries, yet in low- and middle-income countries “the most dispossessed, outcast, powerless group of women in the world” [12] are still at risk of this condition [13]. The uneven global and national distribution suggests a complex picture of determinants contributing to fistula development.

Altogether, the severity of the condition, its consequences, as well as the uneven distribution of OF explain the superlatives that are used to describe its global burden. This scoping review aims to give a critical overview of the determinants of health that contribute to the development of OF in Uganda, to create a better understanding of the process and to give a framework for improvement of health policy.

## Methods

**Fig. 1:**
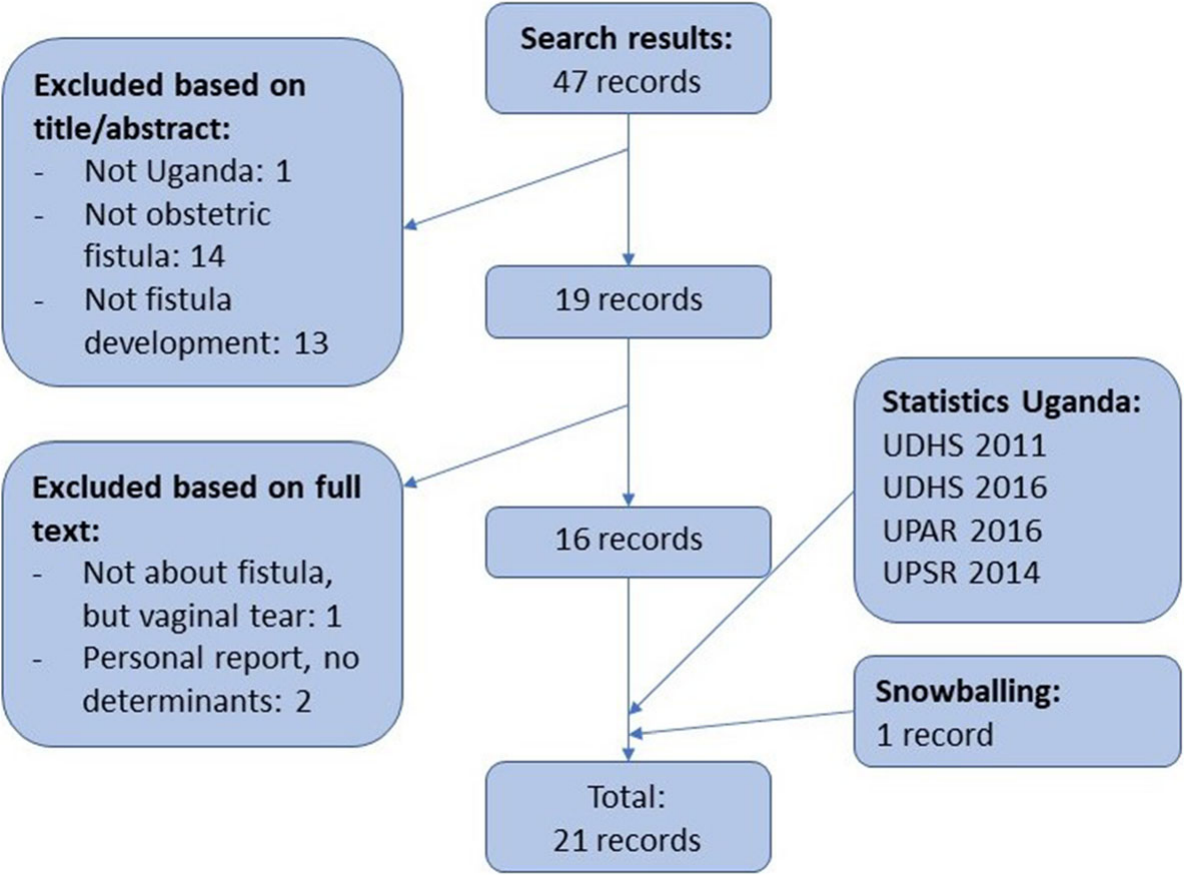
Flow diagram of literature search. Legend: UDHS = Ugandan Demographic and Health Survey, UPAR = Uganda Poverty Assessment Report, UPSR = Uganda Poverty Statement Report

A scoping review of literature was performed in October 2018, using the PubMed/MEDLINE database as well as data from Ugandan DHS (UDHS), the Ministry of Health, the World Bank, and the Ministry of Finance. The key words for the PubMed search were synonyms for ‘obstetric fistula’ and ‘obstructed labour’ (Table 1) in combination with ‘Uganda’. No delimiters were used. The search results were screened by both authors independently. All studies reporting determinants of OF development in Uganda were included. Any disagreements were resolved through discussion between authors and reading of full text. Using the snowballing technique (within PubMed and Google Scholar) one more article was included, not exclusively about Uganda, but describing a pooled analysis of data from SSA, hence not showing up in the original search. The flow diagram of the search can be found in Fig. 1.

**Table 1:** Search terms for the Pubmed/MEDLINE search

Search terms [Title/Abstract]		MeSH terms
vaginal fistula	rectovaginal fistula	vaginal fistula
genital tract fistula	obstructed labour/labor	vesicovaginal fistula
genital fistula	prolonged labour/labor	rectovaginal fistula
vesicovaginal fistula	obstetric fistula

**Fig. 2:**
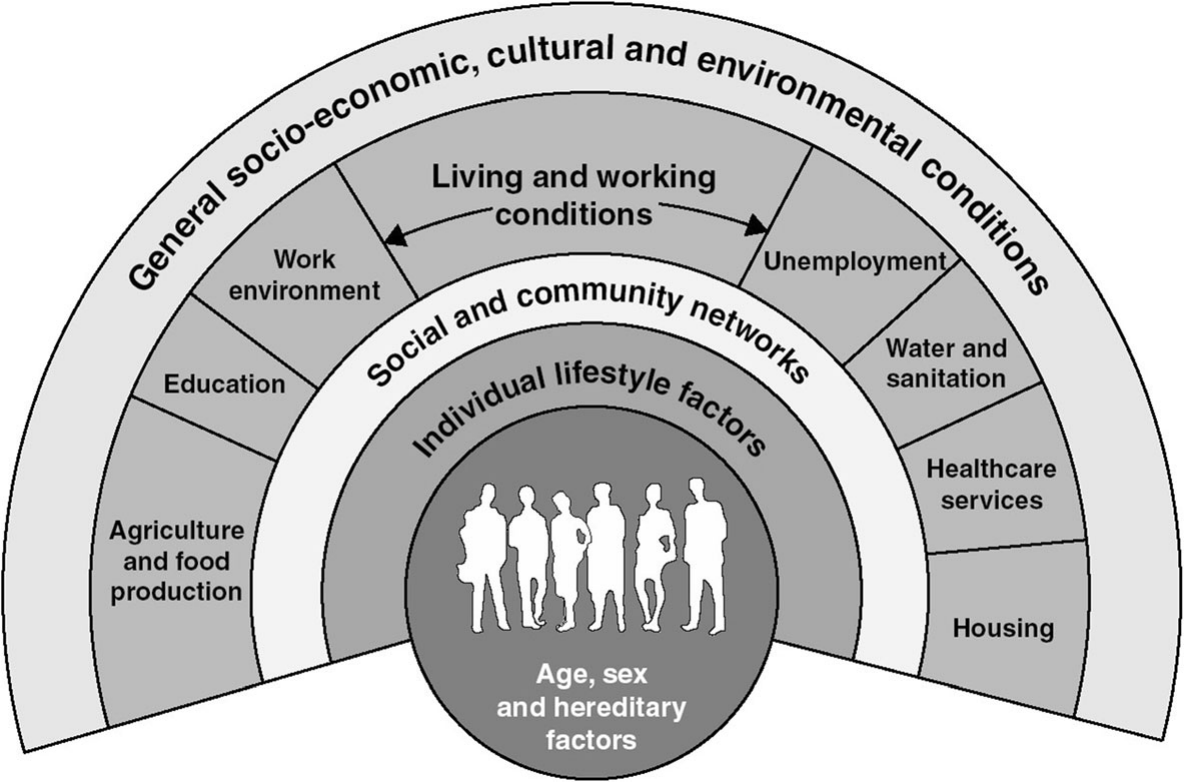
Model for the main determinants of health. Printed with permission of the Institute for Future Studies, Stockholm, Sweden. Source: Dahlgren G, Whitehead M. Policies and strategies to promote social equity in health. Stockholm: Institute for Future Studies; 1991 [14].

Any factors of influence for OF development were extracted from the selected publications. Publications describing determinants of obstructed labour in Uganda were also included, in assumption that these determinants indirectly play part in OF development. The results were analysed and organised using the different levels of the model for the main determinants of health by Dahlgren and Whitehead [14]. This model aims to unravel a wide range of factors that threaten, promote or protect health, and is consequently very suitable to use when formulating interventions within the setting of policy-making [14]. Dahlgren and Whitehead argue that individual lifestyles and attitudes, social and community networks, living and working conditions, as well as overall structural conditions shape a specific health divide resulting in health inequity. The model depicts four levels for policy intervention, and one level that is ‘fixed’ or uncontrollable, namely age, sex and genetics (Fig. 2) [14]. The epidemiological distribution of OF suggests considerable inequity in the presence of determinants of health at different levels of society, thus making this model highly relevant to approach the problem of OF.

Using the model, Ugandan statistics were further analysed to identify possible (indirect) determinants of OF. More literature was sought to explore these factors. The authors reached full consensus about the final presentation of the results.

## Results

The literature search yielded a variety of publications covering determinants of OF in Uganda. These determinants will be reviewed following the layers of the model, though most determinants overlap the different levels.

### Age, sex and constitutional factors

When taken from the greater context, the mechanics of obstructed labour can be narrowed down to a passage that does not match the passenger. Besides malpresentation or malposition of the foetus, the available space or shape of the ‘passage’, the female pelvis, is an important factor for developing obstructed labour and consequently OF.

#### Age

The female pelvis continues to grow until late adolescence, even after the menarche. Consequently, an age below 18 years is associated with an immature pelvis and therefore a higher risk of cephalopelvic disproportion resulting in obstructed labour [15, 16]. At first sight of the ages of the UDHS 2016 respondents, one might mistakenly think OF is a problem of 45–49 year-olds, since the highest percentage of women that have experienced OF is found in that group [11]. However, this is just a reflection of the prevalence of OF, which reflects the cumulative incidence of OF, as well as the low repair rate and barriers to care seeking, not the age at which fistulas are developed.

#### Sex

Fistulas are a complication of obstructed labour which occurs only among women that have had a pregnancy. Therefore, being female is a determinant.

#### Height

Although debated, short stature (women with a height of 150 cm or less) is often indicated as a risk for OF [16, 17]. Even though one could argue that this is a factor shaped by genetics, height is influenced greatly by nutrition and a short stature can thus also be a result of stunted growth due to childhood malnutrition.

#### Race and tribe

African women are believed to have a more narrow pelvis than Europeans, which predisposes them to cephalopelvic disproportion [18, 19]. Within Uganda, the mean pelvic height is significantly different within several tribes, yet regression modelling does not show a strong correlation between tribe and pelvic height [20]. The correlation between certain areas and pelvic height is stronger, which suggests that not so much the genetics of a certain tribe, but more so other determinants linked to a certain area that influence the pelvic size [20].

Although the association remains disputed, several studies in the United States report that Afro-American girls have an earlier menarche than Caucasian girls, even after adjustment for weight, height, and other characteristics [21, 22]. Because early menarche creates the possibility of getting pregnant younger, this suggests that race is an indirect determinant for OF, especially since early menarche is not linked to early maturation of the pelvis [23]. However, age at menarche differs significantly in urban or rural girls in Uganda, and is somehow linked to nutritional status although the nature of the association is still unclear [24]. Thus, this seemingly ‘fixed’ determinant is actually influenced by determinants on multiple levels.

Also, a recent study found that the frequency of OF during the first pregnancy is not increased among women who experience their first pregnancy within 2 years of menarche compared to a first pregnancy later in life [25]. This could suggest that maturation of the pelvis is the important factor and that maturation is related to age more than menarche.

### Individual lifestyle factors

#### Age at first intercourse or pregnancy

In line with determinant of age mentioned earlier, the UDHS of 2006 shows that an older age at first intercourse (20–24 years compared to 15–19 years), often seen as a proxy for age at first pregnancy, is associated with lower presence of OF [26]. Likewise, in the pooled analysis of DHS data of SSA countries, an age below 14 years at first intercourse or at first birth has a positive association with development of OF [16]. A case-control study in Western Uganda does not show a significant difference between women with or without fistula and their age at first pregnancy, nor any association with a young age at marriage [17]. However, it is very likely that the performed frequency matching by age combined with an overall low age at marriage in the study region biased the results [17].

#### Nutrition & smoking

Nutrition plays part at several levels. As mentioned earlier, it affects overall growth, including pelvic growth, and might have an effect on menarche [24]. Also, it is generally accepted that good nutrition is essential for tissue repair and immunity. In this line of argument the development of OF and especially the severity of fistula is related to malnutrition, in the sense that malnourished women have poorer healing after birth trauma [12]. The same might be said for smoking women, although the number of women that smoke is low in Uganda, hence the effect might not be relevant [11]. Malnutrition (and smoking) during pregnancy also negatively impacts on intra-uterine growth of the foetus, which in itself is a predictor for stunted development during childhood, creating a new generation vulnerable for obstructed labour [27, 28]. On the other hand, giving birth to a smaller baby (< 3500 g) is negatively associated with OF, thus malnutrition and smoking might indirectly also be protective of OF [17, 27, 28].

Even though nutrition is a lifestyle factor, it is very much dependent on economic means and employment, education, available food through the local agricultural sector, food production, and even climate change, hence crossing almost all levels of the model [29–32].

### Social and community networks

#### Gender: sexual empowerment

Although intercourse, pregnancy and marriage at a young age can be seen as individual lifestyles, cultural norms and beliefs play an important role when it comes to the position of women and the choices they are able to make. The high unmet need for family planning, limited participation in decision-making, and acceptance of wife-beating are just a few indicators that female empowerment in Uganda is limited [10]. Although knowledge about family planning methods seems widespread in Uganda, limited empowerment is associated with limited use of these methods [10, 33–35].

#### Gender: seeking care

Gender roles also become visible in the decision to seek care. Although not observed in SSA overall, several studies in Uganda including the UDHS 2006 show that women with OF are more likely to indicate that getting permission to seek care was a ‘big problem’ than women without OF [16, 26, 36, 37]. In an Ugandan cohort study among women having fistula repair nearly 1 out of 8 women report that the delay to seek care during labour was due to the family or the husband not allowing her to seek care [38].

#### Sexual or gender based violence

Sexual violence by the intimate partner is common in Uganda [11]. Although sexual violence has been indicated as a determinant of OF, data from the UDHS 2006 do not show a significant association in Uganda [11, 16, 39]. In contrast, a multicounty analysis including Uganda did show an association between sexual or gender based violence and OF [40]. However, the temporality of the association with OF remains unclear. Given the earlier mentioned stigmatization, social and economic consequences of being a fistula patient, it is arguable that the risk of violence increases after the onset of fistula symptoms.

#### Female genital mutilation (FGM)

Depending on the region, in Uganda 0–6,4% of women are circumcised [11]. Because FGM is associated with obstructed labour, the WHO argues that FGM is most likely linked to OF, although there is no research that confirms this association [16, 39, 41–43].

#### Community awareness

A study among fistula patients seeking treatment revealed that 90% of these women identified labour or childbirth as the cause of their VVF [44]. Yet, several other studies show that overall understanding of OF and its causes or its prevention is little and shaped by myths and misconceptions [36, 45–47]. Research among fistula patients as well as sisters of fistula patients showed that the cause was often not known, or sometimes appointed to actions of health care workers or medical procedures [48, 49]. Such low community awareness or even misunderstanding of the cause makes it unlikely for pregnant women or their family to seek care for labour [50].

#### Traditional birth attendants (TBAs)

In Uganda 10,7% of births are assisted by TBAs, of which about 80% are conducted with the use of herbs that can have some oxytocin-like effect, but can also be harmful [11, 51]. Not using herbs during labour had a protective association with OF [17]. Although many TBAs are willing to refer, knowledge about the causes and prevention of OF is limited [52]. Besides not identifying obstructed labour on time, the notion that fistulas are created by manoeuvres and practices of trained health care workers is a barrier to well-timed referrals [52]. However, there was no significant association between presence of an unskilled or skilled birth attendant at delivery and OF [17].

### Living and working conditions

#### Education

Education above primary level of women themselves or their spouse is a protective determinant of OF [17]. In SSA however, being able to read is protective and not so much the post-primary education, which might be a reflection of the actual obtained quality or longer duration of education [16]. Keeping in mind that 1 in 5 women, the majority living rurally, have never had any form of education in Uganda, this becomes highly relevant [11].

#### Socioeconomic status of women

In line with mentioned empowerment, a woman who is the income earner is less likely to be associated with OF compared to when she is a housewife, whereas being a peasant farmer makes her more likely [17]. The profession of the spouse does not have a significant association with the presence of OF [17].

#### Distance to health units

The mean distance to the nearest emergency obstetric care unit is significantly higher among the women with OF (17,5 compared to 5 km) [17].

#### Delays to seek care

The mean time before seeking care from onset of labour is significantly longer in women with OF, as well as the mean duration of labour [17, 38, 53]. Reasons for delay, besides not being able to take the decision to seek care, include the lack of transport, insufficient financial means, or the facility being too far away [36–38, 49, 54, 55].

#### Quality of care

Although not attending antenatal care (ANC) health education classes is a risk factor, ANC attendance irrespective of the number of visits, had no negative or positive association with OF [17]. Birth by Caesarean is strongly associated with OF, which is most likely a reflection of the presence of obstructed labour that is treated by caesarean and the risk of damaging the tissue during this surgery [17]. Additionally, even though the earlier mentioned delays suggest that the damage might already be done before hospital arrival, a substantial part of the observed fistula is due to injuries created by the surgeon [17]. Also, women experienced delays in receiving care when at a health centre, due to shortages of supplies and severe shortages in the number of medical staff [37, 55].

### General socioeconomic, cultural and environmental conditions

In certain regions of Uganda the risk of developing OF is significantly higher compared to other regions, although regional differences between determinants are not always significant [15, 26]. The explanation might be found in the accumulation of determinants: women living in rural areas are overall younger at marriage and first intercourse, have lower education, less wealth, and less power of decision-making [10, 11, 26]. Nevertheless, Uganda’s overall socioeconomic progress and poverty reduction over the last decades is remarkable, mainly due to agricultural income growth driving on peace in northern Uganda, improved regional crop markets, and good weather [56]. Yet because many of the poor are employed in the agricultural sector, they remain especially vulnerable to climatic shocks, pests, and price fluctuations [57]. These factors are therefore also associated with the financial barrier to reach and make use of the already limitedly available health care services in rural areas [56, 57].

## Discussion

This scoping review of literature has rendered several starting points for improvement of health policy. Roughly, the process towards OF development can be divided in two: on the one hand development of obstructed labour, on the other hand the management thereof. Many determinants influence both aspects. For example, a low economic status might result in early marriage and childbearing, associated with obstructed labour, but can also increase delays in getting appropriate care due to lack of funds. Similarly, an increase in female education might result in better nutrition, but it also increases awareness of the necessity of skilled birth attendants during childbirth [58]. Consequently, aiming to improve the socioeconomic status of women in remote areas will theoretically impact both the prevention of obstructed labour, as well as the timely access to care in the case of obstructed labour.

The reviewed research on OF in Uganda covers many aspects of the model presented by Dahlgren and Whitehead, but not all proposed determinants are covered [14]. When further unravelling the determinants that are included in this review, the list extends far beyond what has been mentioned. For example, possible factors of influence could be the distribution of health facilities, the monitoring of labour and interpretation of findings, birth preparedness, fear of caesarean sections, or inadequate post-delivery care. One of the strengths of this review is the inclusion of a broad variety of studies to reflect the diverse set of determinants. However, performing a literature study to map existing determinants does imply the expectation that many determinants have been studied separately. Yet, as this review shows, there are many factors involved that are often intertwined with, dependant of, or reinforced by other factors. It is unlikely that all those complex factors can be studied independently, and it is thus unlikely that this review is fully comprehensive.

Furthermore, a limitation of this study is that for most included epidemiological publications the data of the UHDS of 2012 was analysed, not the more recent UHDS of 2016 published in 2018. Nevertheless, though the overall environment may have changed in Uganda, it is unlikely that the determinants of OF are different between 2012 and 2016. The relative weight or relevance of certain determinants might have shifted, but that is not the scope of this review.

Many reports of risk factors for OF are based on patient characteristics in facility-based studies, leaving limited options to compare the results of this study to risk factors in other countries [59]. There are only a few studies that report DHS data in relation to OF of which a pooled analysis of SSA countries was included in this review [9, 26, 60, 61]. Just like in Uganda, both in Ethiopia as well as in Pakistan, the geographical region and place of residence was a determinant for OF [60, 61]. This suggests that within different countries there is a similar conglomerate of risks factors for OF in specific regions. Also, low educational status, young age at first marriage, and low socio-economic status were significant determinants of OF, similar to the findings in Uganda [60, 61]. Some factors, like the effect of post-primary education or having problems obtaining permission to seek health care, were not found as significant in the pooled analysis of DHS data, but did show to be significant in different national reports [9, 26, 60, 61]. This shows that because of the many different cultural and context-specific factors that are involved in the process of OF development, pooling international data can result in missing the unique factors for a certain country. Similarly, some correlations can seem very strong in a pooled analysis, but might actually be less relevant for a specific country. This underlines the importance of studying the determinants of health within a specific context.

In line with this review, it would be interesting to further investigate the overlap of determinants of OF with determinants of other health conditions. For example, willingness to cervical cancer screening has been linked to a higher level of education, as well as formal forms of employment, and might even show more overlap with OF if further studied [62–64]. This would mean that programs to address these socio-economical determinants could be of interest to several different stakeholders and might show to be very cost-effective. Nevertheless, the focus on socio-economical determinants of health should not surpass focus on health care services. During the decades of the Millennium Development Goals, government interventions like the recruitment of midwifes and the distribution of Emergency Obstetric Care equipment to health facilities, have resulted in impressive gains in maternal mortality and morbidity [65]. Also, though prevention is key, treating those that already have fistula remains of great importance especially considering the enormous impact of OF on the affected women. Fortunately, after the launch of the global campaign to end fistula in 2003, much progress has been made in improving the treatment of OF patients, but the best strategy for OF prevention still remains a difficult topic [66–69].

Although the Determinants of Health model is a tool to create a framework for health policy improvement, it does not make predictions about the single most efficient or cost-effective policy. On the contrary, the identified determinants underline the necessity of a combination of a variety of programs, not only focussed on health-system strengthening but also on diverse population based strategies, like gender equality and fighting malnurtrition [68].

## Conclusion

Early child bearing, childhood malnutrition, low socioeconomic status and empowerment of women, low community awareness of what causes OF and overall lack of access to emergency obstetric care paint the picture of OF in Uganda. Poverty and low education link these determinants, creating a web that is disproportionately hard to escape from for the poorest women. The Ministry of Health should aim to improve health care services of family planning, emergency obstetric care, skilled birth attendance, and obstetric fistula treatment, but even more so cooperate with other sectorial ministries to tackle the determinants that shape the generative environment for OF and limit the access to care. Because all in all, the picture of OF is principally a picture tainted by inequity.

## Data Availability

Data sharing is not applicable to this article as no datasets were generated or analysed during the current study.

## Abbreviations

OF: Obstetric fistula
WHO: World Health Organization
SSA: Sub-Sahara Africa
DHS: Demographic and Health Survey
UDHS: Ugandan Demographic and Health Survey
UPAR: Uganda Poverty Assessment Report
UPSR: Uganda Poverty Statement Report.
FGM: Female genital mutilation
TBA: Traditional birth attendant
ANC: Antenatal care
