# Correction to: Obstetric fistulas in Uganda: scoping review using a determinant of health approach to provide a framework for health policy improvement

**DOI:** 10.1186/s12884-020-02999-5

**Published:** 2020-05-18

**Authors:** Geerte C. den Hollander, Erica W. M. Janszen

**Affiliations:** 1 Maternity and Surgical Departments, Saint Francis Hospital, Mutolere, Kisoro Municipality Council, Kisoro, Uganda; 2 Gynaecology and Obstetrics Department, Kampala Hospital, 6C Makindu Close, Kololo, Kampala Uganda; 3 Gynaecology and Obstetrics Department, OLVG Hospital, location Oost, Oosterpark 9, Amsterdam, the Netherlands

**Correction to: BMC Pregnancy and Childbirth (2020) 20:257**

**https://doi.org/10.1186/s12884-020-02951-7**

Following publication of the original article [1], the authors would like to update the Acknowledgements section as follow:

Acknowledgements

We would like to thank the team of the Netherlands’ Course on Global Health and Tropical Medicine of the KIT Royal Tropical Institute in Amsterdam for passionately teaching the determinants of health model. The lectures and assignments of the course led to the first draft of this paper. Especially the guidance and support of Maaike Flinkenflögel was of great value. We thank professor Florence Mirembe from the Department of Obstetrics and Gynaecology of the Makerere University College of Health Sciences in Kampala, Uganda, for sharing her thoughts after reading our manuscript.
